# Development of Polymer Blends Based on PVA:POZ with Low Dielectric Constant for Microelectronic Applications

**DOI:** 10.1038/s41598-019-49715-8

**Published:** 2019-09-11

**Authors:** Shujahadeen B. Aziz, M. F. Z. Kadir, M. H. Hamsan, H. J. Woo, M. A. Brza

**Affiliations:** 1 Prof. Hameeds Advanced Polymeric Materials Research Lab., Department of Physics, College of Science, University of Sulaimani, Kurdistan Regional Government, Qlyasan Street, Sulaimani, Iraq; 2 Komar Research Center (KRC), Komar University of Science and Technology, Kurdistan Regional Government, Sulaimani, 46001 Iraq; 3 Centre for Foundation Studies in Science, University of Malaya, Kuala Lumpur, Malaysia; 4 Centre for Ionics, Faculty of Science, University of Malaya, Kuala Lumpur, Malaysia; 5 Department of Manufacturing and Materials Engineering, Faculty of Engineering, International Islamic University of Malaysia, Kuala Lumpur, Gombak Malaysia

**Keywords:** Energy science and technology, Materials science, Physics

## Abstract

There is a huge request for the development of low dielectric constant polymeric materials for microelectronic applications. In thisstudy, polymer blends based on PVA:POZ with low dielectric constant has been fabricated. The results of XRD indicate that crystalline domain is enhanced at higher POZ concentration. Brilliant phases between spherulitesare attributed to the enhanced crystalline domains at high POZ content. White portions are appeared in SEM images on the surface of PVA:POZ blends. From EDX analysis, these leaked portions are referred to the POZ material. The number and sizes of the white portions were also found to increase with increasing the POZ content. Using electrical equivalent circuits (EEC), electrical impedance plots (Z″ vs Z′) are fitted for all the samples. The results of impedance study illustrated that the resistivity of the samples increases with increasing POZ concentration. From dielectric measurements, dielectric constant was found to decrease with the introduction of more POZ into the PVA polymer. It is found to be about 1.68 at 40 wt.% POZ. Insulating materials with low dielectric constant (ε′ < 2) are found to be important in the electronics manufacturing, owing to decrease in crosstalk, resistance-capacitance time delay and power dissipation in high-density circuits. Therefore, further investigations concerning the dielectric constant and impedance for all the samples are also carried out. The real and imaginary parts of electric modulus are studied, where minimizing of electrode polarization can be achieved.

## Introduction

Dielectric materials have attracted great attention due to their uses in energy storage applications. Among a variety of physical properties, such as high dielectric constant, low dielectric loss and high electric breakdown strength are the mainly significant parameters that offer valuable information concerning the dielectric materials^1^. The demands for polymer materials have progressively increased with the advancement of science and technology. However, with the development of a new polymer type, several limitations have come up, such as synthesis technology,source of raw material, and cost of production^2^. Mixing polymers is a suitable proposed method to attain the new polymer materials, being able to fabricate materials with properties better to those of the individual constituents. This method is assessed to be fewer time-consuming and high-cost^3^. Hence, researchers have focused their attempts on investigation of polymer blends, in which the respective advantages of each polymers in the blended polymer can be combined. Polymer blends are commonly easier to process and shaping and have excellent flexibility. Polymer blends have extensive application outlooks to mix a variety of polymer matrixes in the modern electronic industry^2^. It has been revealed by the previous studies that the membranes of poly(2-ethyl-2-oxazoline) (POZ) with silver salts is widely applied to olefin/paraffin separation^4–6^. Numerous group studies show that there may be POZ applications, in multiple approaches, for drugs development, anda recent review publications show a growing interest in the use of this polymer^7^. It is documented that bio-based polymer blends based on natural polymershave gained much attention from both industrial and academic investigators. This is due to the certain superior properties, such as biocompatibility and biodegradability that can be adjusted to the particular necessities of a range of applications, such as biomedical, pharmaceutical and packaging areas^8^. Dielectric measurements, such as dielectric constant and dielectric loss,can expose significant information with regard to the chemical and physical properties of polymers. These properties canbe dramaticallyinfluenced by incorporatingother polymers or a dopant to the polymer^9^. Polymeric materials with low dielectric constant can be acquired by two key methods. First, the atoms occupied in the production of dielectric polymers are selected in such a way that the molecular polarizability be diminished. The selections of precursor along with the atoms that have suitable electronegativity can lesser the molecular polarizability. Furthermore, the addition of more C–C building units with small polarizability in the polymer backbone can also cause the dielectric constant lessening. Next, it is noticeable from the polarizationmechanism that a low-density pattern of dielectric material corresponds to a low-density of constituent that contributes to the molecular polarizability^10^. In the area of microelectronic applications, a variety of dissimilar polymer materials with low dielectric constants has been tried. As reported by the literature review, polymeric materials with low dielectric constants (ε′ < 2) are established to be vital for microelectronic circuits. Dielectric constants below 2.5 are specified as “no known solution”^11^. In our previous works, electrical impedance spectroscopy has been verified to be a unique method to study the electrical and dielectric properties of polymer based materials^12–16^. In the present work, a novel approach is offered to fabricate low dielectric polymer film. In this regards, our polymeric samples have been found to exhibit low dielectric constant of ε′ = 1.68 at high frequencies, which can be considered as an innovative method to design low dielectric constant polymers.

## Sample Preparation and Characterization Techniques

### Polymer blend preparation

In this work, polyvinyl alcohol (PVA) and poly(2-ethyl-2-oxazoline) (POZ), procured from Sigma-Aldrich, have been used as raw materials for the preparation of polymer blend filmsby using the solution cast technique. For this purpose, 1 gm of PVA was dissolved in 40 mL of distilled water at 80 °C. After cooling to room temperature, the solution was then mixed withdifferentpercentages of dissolved POZ, ranging from 10 wt.% to 40 wt.%. To obtain homogeneous solutions, the mixtures were continuously stirred for 2 hours using a magnetic stirrer. The samples were coded as PVOZ1, PVOZ2, PVOZ3 and PVOZ4, respectively. The mixtures were then cast into different clean and dry plastic Petri dish and left to dry at room temperature until solvent free films were obtained. For further drying, the films were kept in desiccators with blue silica gel desiccant.

### Characterization techniques

X-Ray Diffraction (XRD) patterns of the samples were recorded on a PANalytical Empyrean X-ray diffractometer, operating at 40 mA and 40 kV. The X-ray beam was a monochromaticCuKα radiation (wavelength, λ = 1.5406 Ǻ)and directed under glancing angles in the range of 5° ≤ 2θ ≤ 80° inastepsizeof0.1°. The surface morphology of the films was observed by optical microscopy (OM, MEIJI, model). The photos at a fixed magnification were taken through attached camera-controlled (DinoCapture) software. Furthermore, the surface morphology of the samples was also observed by scanning electron microscope (SEM, Quanta 200 FESEM).

The electrical impedance spectroscopy (EIS) measurement was carried out for the films using an HIOKI 3531 Z LCR Hi-tester at ambient temperature inthe frequency range of 50 Hz to 1000 kHz. The LCR meter was attached to a computer based data acquisition software, through which the real and imaginary parts of the impedance could be calculated. The polymer blend films were cut into discs with diameter of 2 cm and sandwiched between 2identical circular stainless steel electrodes under spring pressure to ensure a good contact.

## Results and Discussion

### XRD analysis

**Figure 1:**
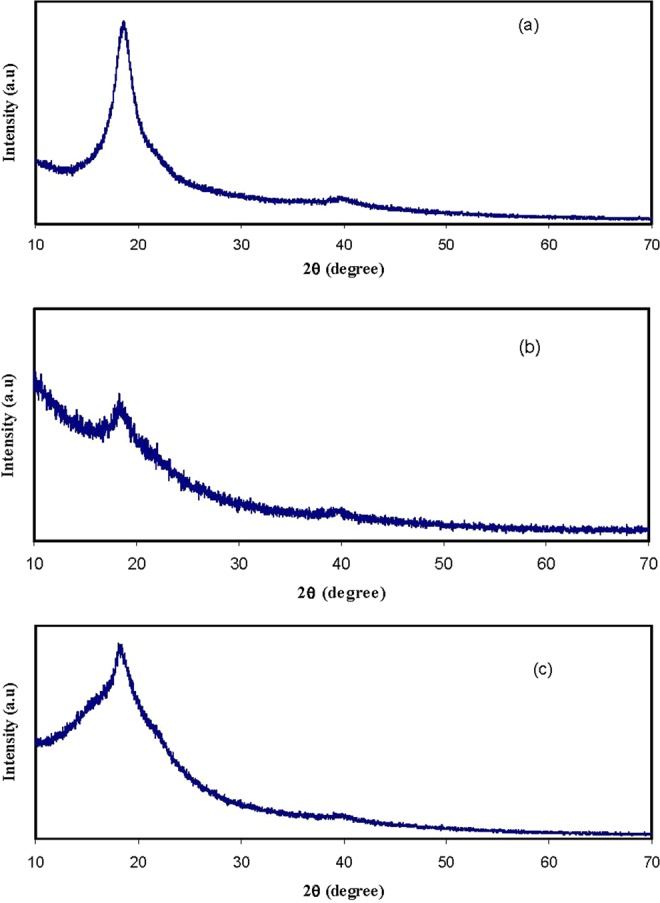
XRD pattern for (**a**) PVOZ0 (pure PVA), (**b**) PVOZ1 and (**c**) PVOZ4 blends.

**Figure 2:**
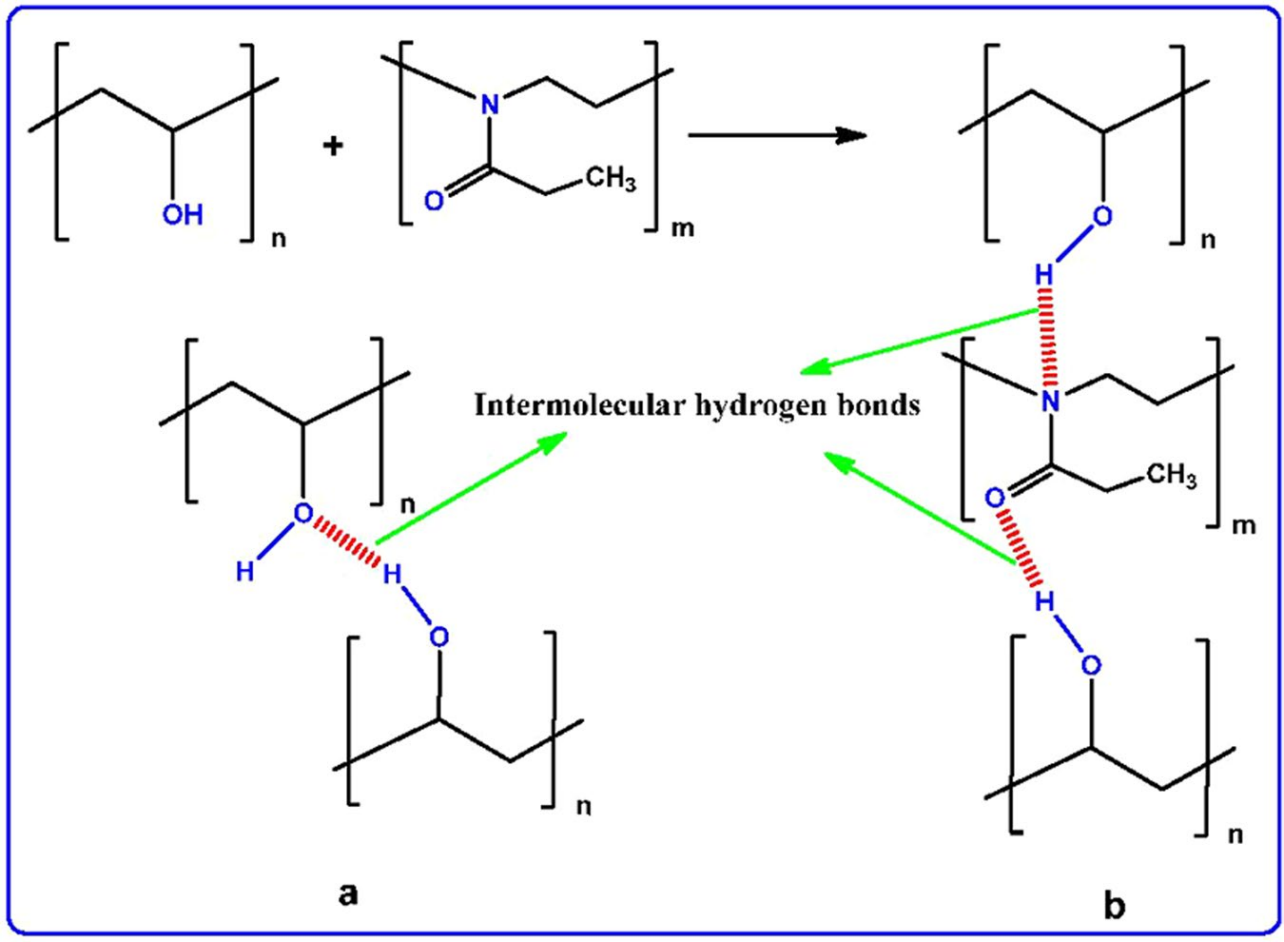
Schematic illustration of hydrogen bonding (**a**) between PVA monomers and (**b**) between PVA and POZ monomers.

Figure 1(a–c) show the XRD patterns for pure PVA and PVA:POZ blend samples. It can be seen from Fig. 1(a) that the XRD pattern shows a sharp and broad peaks around 2θ = 20° and 40° corresponding to crystalline domain and semicrystalline nature of pure PVA, respectively^17,18^. Clearly, it is signifying that the pure PVA includes great quantity of crystalline domain. It is interesting to observe from Figure 1(b) that at 10 wt.% of POZ, the wideness of the main peak of PVA is improved, signifying that the fractional crystallinity of PVA is diminished. Such increase in the wideness and fully vanishing of the peak at 2θ° = 20° can be taken as an evidence for the increase of amorphous state^19,20^. These results can be explainedfrom the perspective of Hodge *et*. *al*.^21^ criterion, in which a correlation between the height of peak intensity and the degree of crystallinity have been established. They reported that as the amorphous fraction increases with the addition of the dopant, the intensity of the XRD pattern decreases^22^. The increase in the non-crystallinityin the system causes a reduction in the energy barrier to the segmental motion of the polymer chains and segments^23^. Therefore, our XRDanalysesclearly confirm the occurrence of complexation between the PVA and POZ polymers. The crystalline peak of PVA is improved again at 40 wt.% of POZ, which can be ascribed to the enhancement of hydrogen bonding at higher concentration of POZ. The raise of hydrogen bonding at 40 wt% of POZ is shown schematically in Fig. 2. It is clear from Fig. 2(a,b) that the intermolecular hydrogen bonding develops into double when more POZ added to PVA. In this case,hydrogen bonding increases. Thus the enhancement of hydrogen bonding is responsible for the enhancement of the crystalline peak. It is well well-known that the molecular polarizability lessens in the crystalline polymeric materials, while a large degree of molecular polarizability can be gainedin amorphous phases^10^. In later sections, further studies of electrical properties, such as dielectric properties, have been given and correlated with our XRD results.

### Morphology study

**Figure 3:**
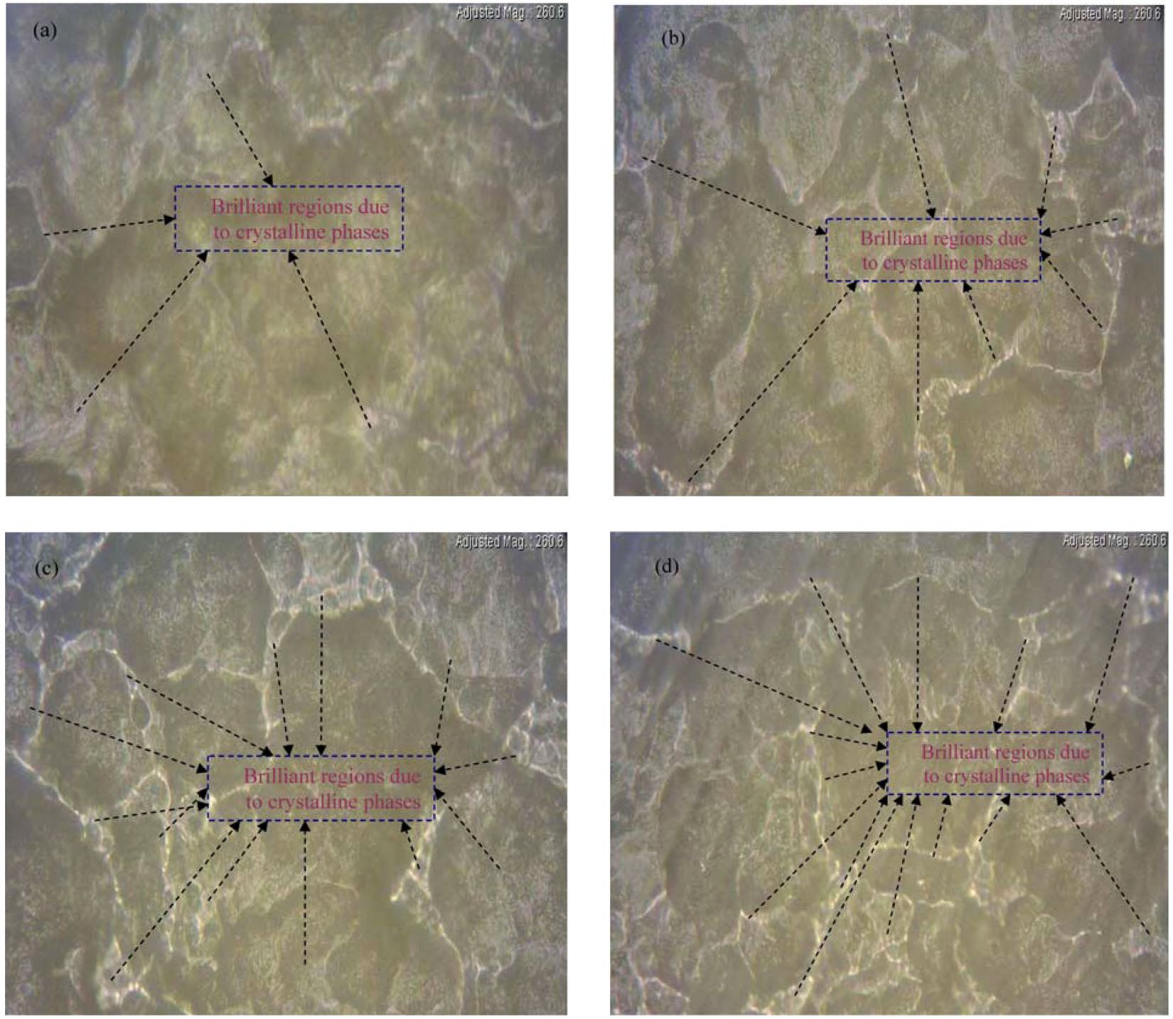
Optical microscope images for (**a**) PVOZ1, (**b**) PVOZ2, (**c**) PVOZ3 and (**d**) PVOZ4 blend samples.

Previous studiesoutcome established that when two miscible polymers are mixed together, dissimilar morphologies, such as sphere and lamellar, may appeared depending on the capability of the polymer components in the blend to interact altogether^24^. Figure 3 shows optical microscopic images of the surface ofthe PVA:POZ blend samples. It is obvious from the images that the dark regions (orspherulites) are attributed to amorphous phases, while the brilliant phases between the spherulitesare ascribed to crystalline domains. Recent studies revealed that optical microscopy could be an efficient technique to observe the occurrence of structural changes within the polymer composites and polymer blend systems^25–28^. A close inspection exposes that the spherulites include numerous small brilliant regions. It is noticeable from the images that at 10 wt.% POZ, the dark regions are lessened and noticeable brilliant phases are emerged. This can be attributed to the enhancement of the crystalline phases. Other researchers have also employed OM technique to examine the crystalline and amorphous phases in PEO based composites. They have ascribed the brilliant spherulites to the crystalline structure and the dark regions to the amorphous phase. The dark boundaries between the spherulites are typically assigned to the presence of amorphous phase^29^. But in the current work the brilliant regions are ascribed to the crystalline phase and the dark spherullites are ascribed to the amorphous domain. This is related to the fact that PEO almost crystalline polymer while PVA contains enormous amorphous phases. Therefore, dark spherullites are attributed to the amorphous regionin PVA. Our elucidation is closely in agreement with OM images and it can be observed that with rising POZ concentration, the brilliant regions also enhances. Therefore, the outcomesacquired in OM technique analysis robustly support our XRD patterns.

**Figure 4:**
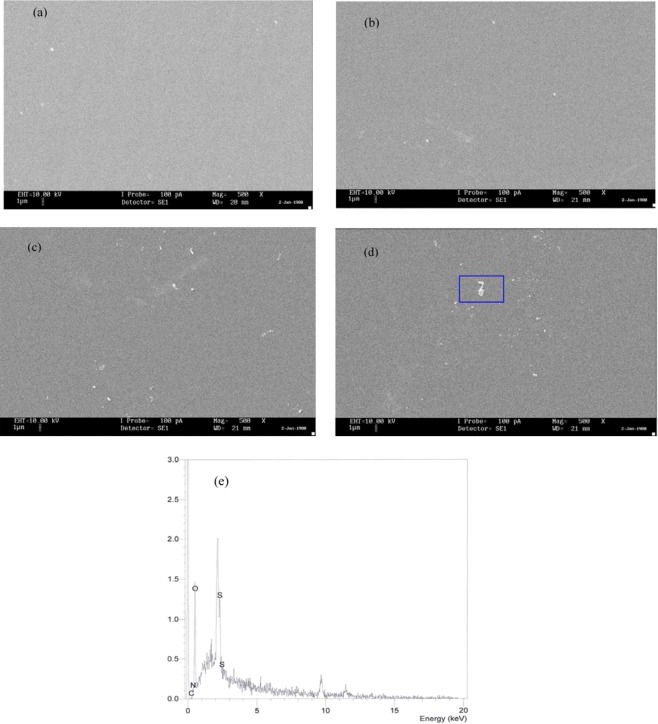
SEM micrograph for (**a**) PVOZ1, (**b**) PVOZ2, (**c**) PVOZ3, (**d**) PVOZ4 blend samples and (**e**) EDX spectra for the white spot inside the blue box.

To support the resultsof XRD and OM analyses, SEM images have been taken for the PVA:POZ blend films. The SEM technique has been established to be a significant tool for investigating the structure and surface morphology of the polymer combines and composites^30,31^. The SEM technique has an extensive range of magnification, permitting us to center of attention merely on a specific area of the film^32^. It is significant, here, to distinguish that the surface morphology and structure of the polymer films are the main properties to sort out their performance. The SEM images were recorded at magnification of 500 x for each sample. Prior to the examination, the films were fixed to an aluminum holder with a conductive tape and subsequently layered with a narrow coat of gold. In our previous work, SEM technique has been employed to identify crystalline structures (referring to the ion diminution and creation of metallic particles) in chitosan:AgNt solid electrolyte system. The SEM results were also used to elucidate the decrease in direct current (DC) conductivity^32^. The SEM technique has also employed by other researchers to identify the protruded salt crystalline structures in polymer electrolytes at high concentrations of the salts^33,34^. Our recent studies exposed that SEM analysis to investigate the happening of phases separation in CS:PEO based polymer blends is a novel approach^31^. Hence, based on these earlier examinations it is probable to say that SEM technique is an appropriate method to investigate a variety of phenomena in polymers, such as ion lessening, ion association and phase separations^30–34^. Figure 4 shows the SEM micrographs for the blended samples. One can discern that some white agglomerations are emerged on the samples surface and their size and number rose with raising POZ concentration. The EDX analysis has designated that these leaked portions are as a result of the POZ material. Results expose that the SEM technique can also be a valuable and efficient approach to recognize the phase separation in polymerblend materials. The manifestation of large number of phase separation is an evidence for the existence of large number of crystalline phases, as established in XRD results.

### Impedance study

**Figure 5:**
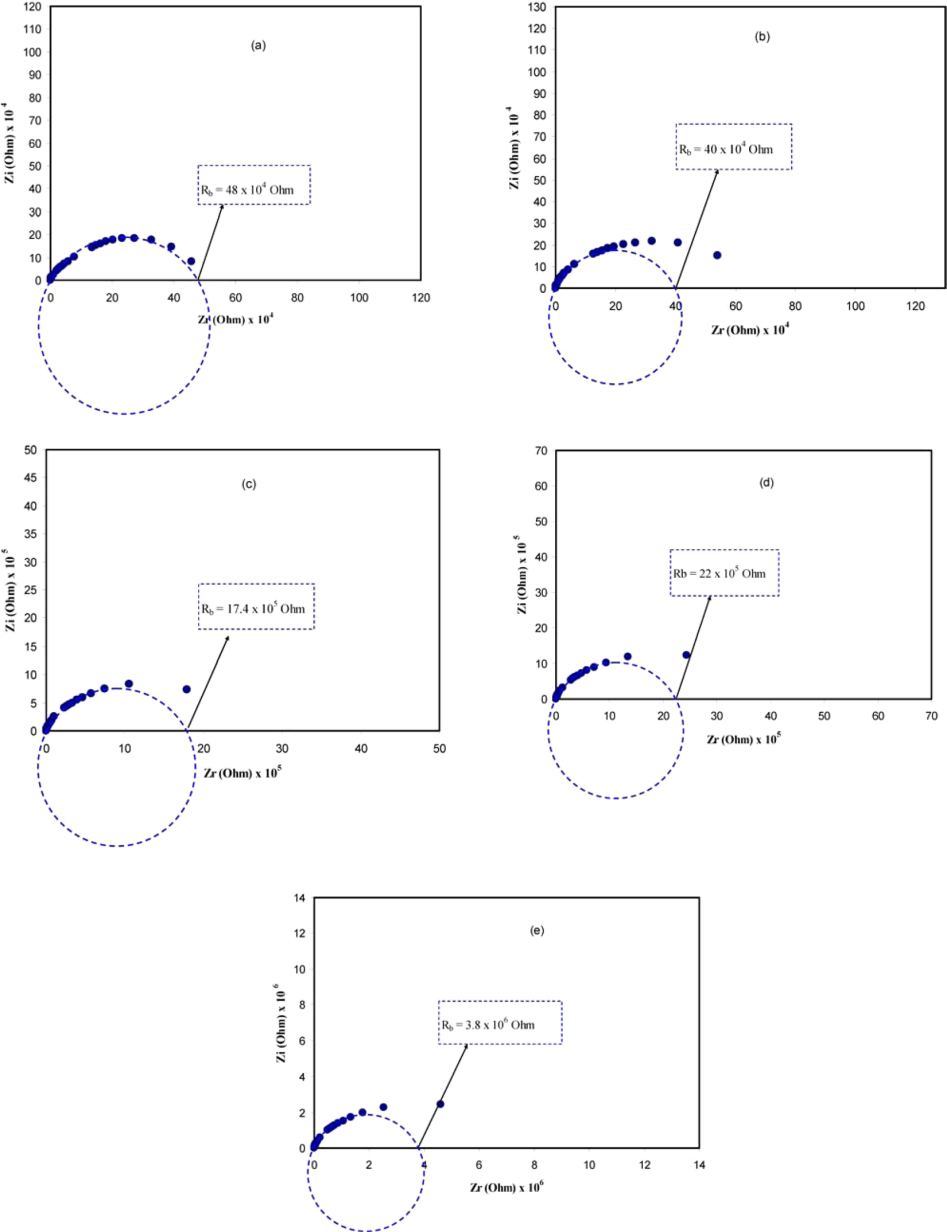
Impedance (Nyquist) plots for (**a**) PVOZ0, (**b**) PVOZ1, (**c**) PVOZ2, (**d**) PVOZ3, and (**e**) PVOZ4 blend samples.

Figure 5(a–e) show electrical impedance plots, complex impedancereal and imaginary parts, i.e., Z′ vs Z″, for the PVA:POZ blend films at room temperature. Impedance spectroscopy is employed to establish the conduction mechanism, examining the presence of polymer chains, mobility and carrier creation processes. From the bulk resistance (R_b_) acquired by the intercept with the real axis of the impedance plots, the conductivities of the polymer complexes can be estimated^35^. It can be seen from Fig. 5 that the plots exhibit a low and high frequency semicircle regions, which are arising from the effect of blocking electrodesknown as electrode polarization (EP) and the bulk effect of the solid electrolyte, respectively. The EP phenomenon originates from the formation of electric double layer (EDL) capacitances through the free charge build-up at the interface between the solid electrolyte and electrode surfaces within polymer electrolytes^36,37^. In order to verify that these data points are linked to the EP effect, theplots of the experimental impedancewere fitted with electrical equivalent circuits (EEC) as can be presentedin later section. At low frequency region, the plots of the complex impedance be required to indicate a straight line parallel with the imaginary axis, i.e., the angle of inclination of the straight line be supposed to be 90°. Though, at the blocking electrodes, the blocking double-layer (i.e., EP phenomena) induces such inclination^38,39^.

**Figure 6:**
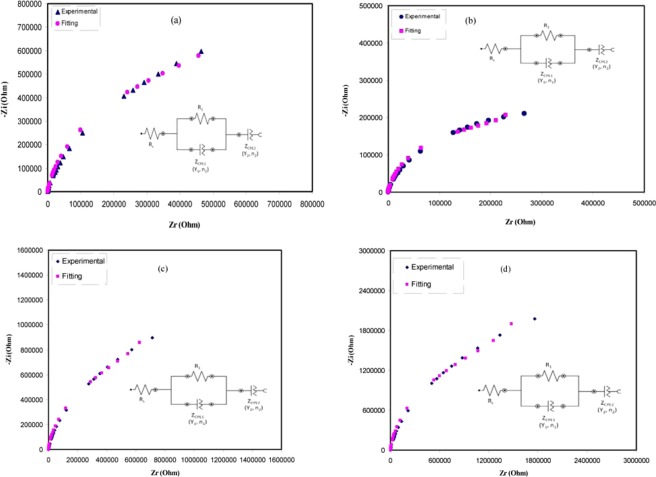
Experimental and fitting (EEC) Impedance (Nyquist) plots for (**a**) PVOZ1, (**b**) PVOZ2, (**c**) PVOZ3, and (**d**) PVOZ4 blend samples. The insets show the electrical equivalent circuits (EECs).

The EEC is typically used for the impedance spectroscopy study, for the reason that it is simple and an entire picture of the system can be presented^40^. The acquired impedance plots can be elucidated regarding the equivalent circuit relating bulk resistance (R_b_) for the carriers in the sample and couple constant phase elements (CPE1 and CPE2) as shown in insets of Fig. 6. The high frequency region signifies the combination of R_b_ and CPE1, whereas the low frequency region signifies CPE2, i.e., the produced double layer capacitance between the electrodes and solid polymer electrolytes. In real system, the CPE expression is more frequently used in equivalent circuit rather than ideal capacitor. This is due that the real solid polymer electrolyte behavior is dissimilar from that of an ideal capacitor concerned in an ideal semicircular pattern^41^.

The impedance plotsare usually appeared as a result of electrical reaction of the material once subjected to an AC signal, which can be equivalent to the electrical circuit model^26,28,31,42^. The EEC cantherefore be used to illustrate the behavior of the dipoles and carriers responses of the considered polymer^26,28,43^. Figure 6(a–e) shows the experimental impedance plots for the polymer blend samples with their equivalent circuits. Since the complex impedance plots shown in Fig. 5 be composed of a quantity of data points and the depressed semicircle at low and high frequencies, correspondingly, the equivalent circuit therefore illustrated by a parallel connection of R_b_ and CPE or bulk capacitance (Z_CPE_) in series with one more CPE initiating from the tilted spike region. The CPE is employed rather than a capacitor to clarify the depressed semicircle^42^. The impedance of Z_CPE_ can be written as:^26,28,43^

1 $${Z}_{CPE}=\frac{\cos (\frac{{\rm{\pi }}{\rm{n}}}{2})}{{Y}_{m}{\omega }^{n}}-j\frac{\mathrm{Sin}(\frac{{\rm{\pi }}{\rm{n}}}{2})}{{Y}_{m}{\omega }^{n}}$$

where Y_m_ is the CPE capacitance, ω is the angular frequency and *n* is corresponding to the deviation of the plot from the vertical axis in complex impedance plots. Here, the real (Z′) and imaginary (Z″) values of the complex impedance (Z*) associated with the equivalent circuit (inset of Fig. 6(a–d)) can be expressed as:

2 $${Z}_{r}={R}_{s}+\frac{{R}_{1}+{R}_{1}^{2}{Y}_{1}{\omega }^{n1}\,\cos (\frac{\pi {n}_{1}}{2})}{1+2{R}_{1}{Y}_{1}{\omega }^{{n}_{1}}\,\cos (\frac{\pi {n}_{1}}{2})+{R}_{1}^{2}{Y}_{1}^{2}{\omega }^{2{n}_{1}}}+\frac{\cos (\frac{\pi {n}_{2}}{2})}{{Y}_{2}{\omega }^{{n}_{2}}}$$

3 $${Z}_{i}=\frac{{R}_{1}^{2}{Y}_{1}{\omega }^{{n}_{1}}\,\sin (\frac{\pi {n}_{1}}{2})}{1+2{R}_{1}{Y}_{1}{\omega }^{{n}_{1}}\,\cos (\frac{\pi {n}_{1}}{2})+{R}_{1}^{2}{Y}_{1}^{2}{\omega }^{2{n}_{1}}}+\,\frac{\sin (\frac{\pi {n}_{2}}{2})}{{Y}_{2}{\omega }^{{n}_{2}}}$$

Table 1 lists all the parameters attained by fit for the plots of impedance with equivalent circuits. The value of R1 in equivalent circuits corresponds to the bulk resistance (R_b_) as revealed in the insets of Fig. 6. It is appealing to perceive that the R_b_ attained from the intersect of semicircles with the real axis of the impedance plots in Fig. 5 are close enough to the values of R1 attained from EEC models as revealed in Table 1.

**Table 1:** The parameters of the circuit elements of the blend films at ambient temperature.

Sample	*R*_1_	*Y*_1_	*n*_1_	*R*_*s*_	*Y*_2_	*n*_2_
PVOZ 1	6.9 × 10^5^	8 × 10^−10^	0.91	17	2.1 × 10^−9^	0.932
PVOZ 2	2.6 × 10^5^	1.4 × 10^−9^	0.906	6.5	6.19 × 10^−9^	0.9301
PVOZ 3	8.4 × 10^5^	6.5 × 10^−10^	0.9103	8.96	1.2 × 10^−9^	0.956
PVOZ 4	1.76 × 10^6^	3.16 × 10^−10^	0.9218	19.88	7.38 × 10^−10^	0.947

### Dielectric properties

**Figure 7:**
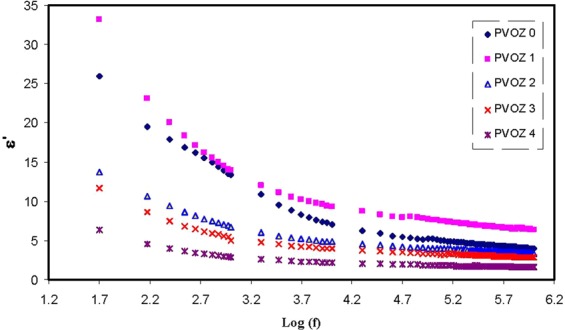
Dielectric constant versus frequency at ambient temperature for pure PVA and PVA:POZ blend samples. Clearly with increasing POZ concentration the *ε*′ decreases.

**Figure 8:**
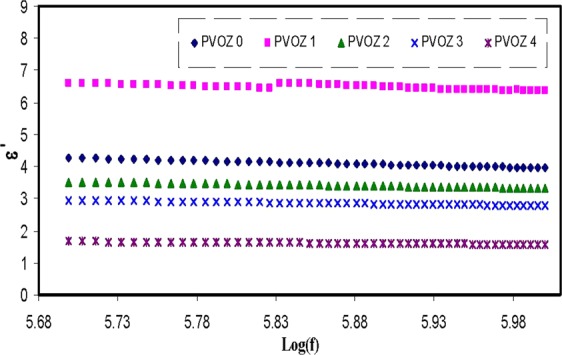
Dielectric constant (high frequency region) versus frequency at ambient temperature for pure PVA and PVA:POZ blend samples. Clearly at 40 wt.% of POZ concentration the *ε*′ drops to minimum value.

**Figure 9:**
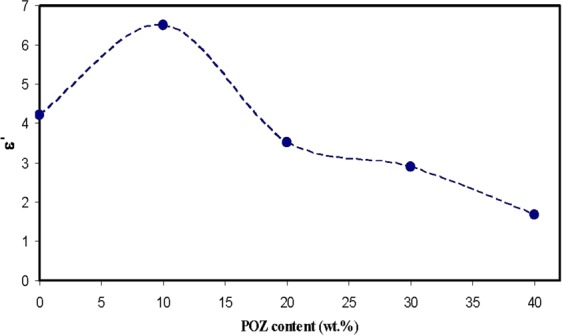
Dielectric constant (at 1 MHz) versus POZ concentration at ambient temperature.

Recent studies revealed that there are numerous methods for characterizing dielectricmaterial properties, such as relative permittivity and losstangent,microwave reflection coefficient, port coaxial and waveguide cells, split post dielectric resonance technique, open-ended probesand terahertz (THz) metamaterials in the THz frequency ranges. In recent years, alot of research activities have been aiming at improvingthe accuracy and sensitivity of material characterization^44–47^. In particular; impedance measurement at various frequencies are found to be an accurate technique to probe the molecular motion of dielectric materials at different temperatures^12,15,16,26,28,48^. The analyses of dielectric relaxation are considered incredibly helpful in comprehending the polymers and their blends performance. A wide frequency range measurement of dielectric relaxation spectroscopy has been carried out to investigate the dipole relaxation in the polymer materials^26^. By using the real (Z′) and imaginary (Z″) part of the complex impedance (Z*), the real and imaginary parts of the complex permittivity (ε*) and complex electric modulus (M*) will beestimated, by means of the following relations^12,15,16,26,28,48^,

4 $$\varepsilon ^{\prime} =\frac{Z^{\prime\prime} }{\omega {C}_{0}({Z^{\prime} }^{2}+{Z^{\prime\prime} }^{2})}$$

5 $$\varepsilon ^{\prime\prime} =\frac{Z^{\prime} }{\omega {C}_{0}({Z^{\prime} }^{2}+{Z^{\prime\prime} }^{2})}$$

6 $$M^{\prime} =\frac{\varepsilon ^{\prime} }{({\varepsilon ^{\prime} }^{2}+{\varepsilon ^{\prime\prime} }^{2})}=\omega {C}_{0}Z^{\prime\prime} $$

7 $$M^{\prime\prime} =\frac{\varepsilon ^{\prime\prime} }{({\varepsilon ^{\prime} }^{2}+{\varepsilon ^{\prime\prime} }^{2})}=\omega {C}_{0}Z^{\prime} $$

where *M*′and *M*″ referto the real and imaginary parts of complex electric modulus and ε′ and ε″ refer to dielectric constant and dielectric loss, respectively. Here, C_o_ indicates the vacuum capacitance of the measurement cell, which can be fromε_o_A/t (where *t* and *A*refer to the thickness and area of the film, respectively), and ω = 2π*f* is the angular frequency, *f* is the frequency in hertz. Figure 7 illustrates the achieved dielectric constant spectra versus frequency for pure PVA and PVA:POZ blend samples. A close inspection of Fig. 7 indicates that with the inclusion of more POZ groups into the PVA polymers, the dielectric constant can be lessened to around 1.9 at 40 wt.% POZ. Figure 8 illustrates the dielectric constant for every sample at high frequency region. The intersections of the lines of Fig. 8 were used to estimate the dielectric constant of the samples at high frequency region. The dielectric constant of PVA versus POZ concentration was shown in Fig. 9. It is noticeable that at 40 wt.% of POZ, the ε′ has decreased to the lowest value (ε′ = 1.68). A previous study exposed that a polymer dielectric constant is mostly described by two parameters^49^. First is the molecular polarizability, which can be altered by changing the nature and number of polarizable groups. Next is the free volume associated with the polymer. Over the recent decades, performance improvements in microelectronic integrated systems have been achieved by raising the speed of the transistor, extra lessening transistor dimensions and compress more transistors onto a microchip. Insulating materials with low dielectric constant (low-ε′) are critical in electronics industrytoday^50^. Particularly, there is a desperate need in high-performance polymers with low dielectric constant and loss factor, because they hold the promise of overcoming the line-to-line crosstalk noise, signal delays and power dissipation in integrated circuits (ICs)^51^. In accordance with the Semiconductor Industry Association (SIA) roadmap, the lowest feature size was targeted to attain 0.15 µm by the year 2001, which essential a dielectric material of ε′ = 2.3 to be attained for their insulation. Thus, for up-coming ICs productions, an ultra small dielectric material (ε′ < 2) will be extremely required^52^. Previous researches have reported that enlargement of spherulite has a reasonably slight impact on the conductivity behavior. However, it is demonstrated that as the crystallites commence to cover a great fraction of the polymer, the conductivity and dielectric constant are enormously lessening^53^. The foremost plausible clarification for the dielectric constant diminishing is the structural densification, i.e., the increase of crystalline arrangement between the presented lamellae. Hence, this leads to the charge carrier transfer to be hindered in the rest of amorphous phase. In such a compact structure, a tiny change of amorphous phase content can create a disruption in stability of the conduction pathway. Alternatively, the lessening of contact with electrodes and the sample stiffness can be the cause for such great conductivity fall or amplify in resistivity^54^.

**Figure 10:**
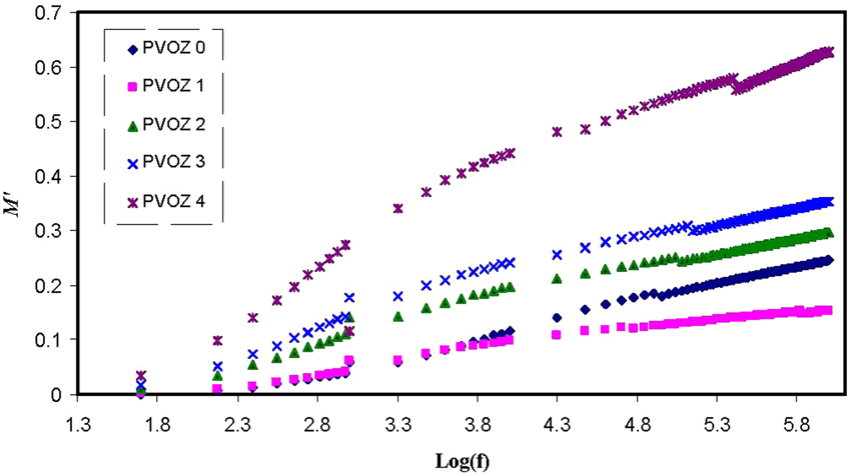
Real part (*M*′) of electric modulus versus frequency at ambient temperature for pure PVA and PVA:POZ blend samples. Clearly with increasing POZ concentration the *M*′ spectra shift to above.

**Figure 11:**
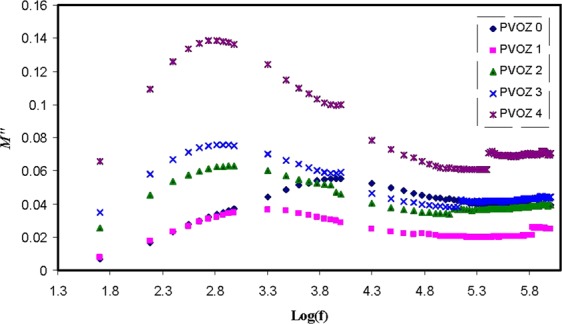
Imaginary part (*M*″) of electric modulus versus frequency at ambient temperature for pure PVA and PVA:POZ blend samples. Clearly with increasing POZ concentration the *M*″ peak shifts higher frequency.

**Figure 12:**
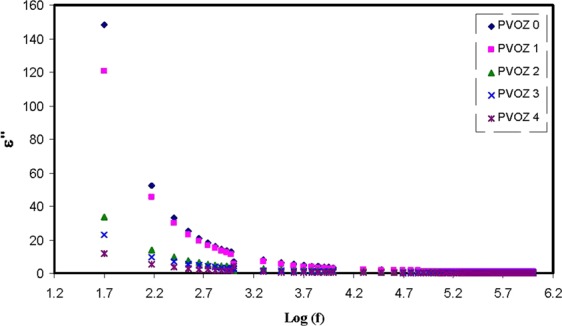
Dielectric loss versus frequency at ambient temperature for pure PVA and PVA:POZ blend samples. Clearly with increasing POZ concentration the *ε*″ decreases.

Currently, the illustration of M* is frequently employed to examine ionic conductivities in link with the correlations of the ionic process with conductivity relaxation time^55^. Explanation of relaxation phenomena throughout the electric modulus formalism offers a number of benefits over permittivity and conductivity relaxation treatments, since it avoids the great difference in the values of ε′ and ε″ at low frequencies and high temperature. Furthermore, complicatedness presenting in the dielectric spectrum investigationcan be surmount by ignoring the addition of space charges and absorbed impurities^56^. Figure 10 shows that at low frequency region, the *M*′ value approaches to zero due to the high capacitance value of the double layer charges^57,58^. Compared with the dielectric constant pattern, the *M*′ spectra appears to be precisely in dissimilar way. The high value of dielectric constant was seen at low frequency as revealed in Fig. 7. The electric modules (*M*′ and *M*″) exhibit a lowest value at high frequency, because they are reciprocal with the complex dielectric constant. Figure 11 shows the imaginary part of modulus spectra. Here, the peaks of the conductivity relaxation are identified. It can be seen that with increasing POZ content, the relaxation peak shifts to lower frequency side andthis indicates an increase in relaxation time (τ_o_ = 1/ω_max_) extends. The raise of relaxation time is linked to the reduction of segmental mobility in the blend samples amorphous phase. These peaks are the transition regions between the translational extensive range ionic movement (translational mobility) and small range segmental movement (dipolar mobility), i.e., at elevated frequency range, the carriers are confined to potential wells and shiftingthrough a small distance^14,59^. The low frequency side of the peaks is the region where the molecules and dipolesget enough time to reorient themselves with the alternating electric field and polarization occurs. Accordingly, they will create a double capacitance layer between the electrode-electrolyte films. This creates a high dielectric constant and thereforean extremely small of M″. On the contrary, the high frequency side of the peak is determined to be the region where the ions can merely perform local (re-orientation) movement^60,61^. The existent of peak in *M*″ spectra and missing of peak in ε″ (see Fig. 12) demonstrate that the motion of carriers or molecules is robustly coupled with the polymer segmental motion^61,62^. It is obvious from the *M*″ plot that the peaks on both sides of the maxima are asymmetric and thus it cannot be anticipated by ideal Debye manner^62^.

## Conclusions

In this work, polymer blend films based on PVA:POZ with low dielectric constant have been fabricated. The XRD outcome indicatesthat the crystalline domain enhances at higher POZ content due to the increase of hydrogen bonding. The detected brilliant phases from the OM study between spherulites are established to be as a resulting of the improved crystalline phase. White portions were emergedin SEM images on the film surface, which enhanced in number and size with the increase of the POZ content. Outcomes from the EDX analysis illustrate that the leaked portions are ascribed to POZ material. Electrical impedance plots for the samples are fitted with electrical equivalent circuits. The impedance study demonstrated that the resistivity of the samples increases with increasing POZ concentration. The dielectric constant investigation illustrates that introducing of more POZ into the PVA polymer can lessen the dielectric constant to around 1.68 which is crucial from the viewpoints of the electronics manufacturing, owing to decrease in crosstalk, resistance-capacitance time delay and power dissipation in high-density circuits. Further examinations regarding the dielectric constant and impedance for all the samples were also carried out. For all the blend samples, the real and imaginary parts of electric modulus were investigated. In the electric modulus investigation, the electrode polarization could be lessened and consequently the low dielectric constant material spectra appeared above the other curves. The peaks in M″ plot exhibits asymmetric shape on both sides of the maxima and thus it cannot be anticipated by ideal Debye model.

## Data Availability

Even all data from the study and the findings are contained within the paper; any more information can be accessible from the corresponding author upon request.
